# Electronic Clinical Decision Support Tools to Manage Patients with Lower Respiratory Tract Infection: Clinicians’ Perspectives in Sri Lanka

**DOI:** 10.4269/ajtmh.24-0576

**Published:** 2025-03-11

**Authors:** Warsha De Zoysa, Dhammika Palangasinghe, Champica Bodinayake, Ajith Nagahawatte, Jayani Gamage, Maria D. Iglesias-Ussel, Stefany Olague, Christina Galdieri, Ruvini Kurukulasooriya, Senali Weerasinghe, Madureka Premamali, James Ngocho, Armstrong Obale, Hrishikesh Chakraborty, Truls Ostbye, Susanna Naggie, Christopher W. Woods, Evan Myers, Melissa H. Watt, L. Gayani Tillekeratne

**Affiliations:** ^1^ Department of Medicine, Faculty of Medicine, University of Ruhuna, Galle, Sri Lanka;; ^2^ Duke-Ruhuna Collaborative Research Centre, Faculty of Medicine, University of Ruhuna, Galle, Sri Lanka;; ^3^ Duke Global Health Institute, Duke University, Durham, North Carolina;; ^4^ Department of Microbiology, Faculty of Medicine, University of Ruhuna, Galle, Sri Lanka;; ^5^ Department of Medicine, School of Medicine, Duke University, Durham, North Carolina;; ^6^ Duke Clinical Research Institute, Duke University, Durham, North Carolina;; ^7^ Kilimanjaro Christian Medical Centre, Moshi, Tanzania;; ^8^ Department of Family Medicine, Duke University, Durham, North Carolina;; ^9^ Department of Population Health Sciences, School of Medicine, University of Utah, Salt Lake City, Utah

## Abstract

In low-resource settings, providers often manage lower respiratory tract infections (LRTIs) without diagnostic tests, which may cause antibacterial overuse. Electronic clinical decision support tools (eCDSTs) can support evidence-based decision-making and judicious use of antibacterials. This study aimed to explore the potential of an eCDST to help providers in Sri Lanka effectively manage LRTI. Semi-structured interviews were conducted with 15 clinicians, including 10 males and five females, with an average of 11.6 years (range: 4–25 years) of clinical practice. The interview guide covered clinicians’ interest in an eCDST to manage LRTI and their feedback regarding the desired features of such a tool. Interviews were audio-recorded, transcribed, and coded for themes related to: interest in an eCDST for LRTI, desired tool capabilities, development concerns, and tool design characteristics. All expressed interest in incorporating eCDSTs into their practice. However, the majority emphasized that clinical judgment must supersede recommendations from an eCDST. Four themes emerged regarding desired tool capabilities: information about the pathogen, treatment recommendations, severity of the LRTI, and monitoring of patient progress. Six themes emerged regarding tool development considerations: validated algorithms, regional specificity, seasonality, inclusion of patient’s risk factors, scalability, and the importance of updated and locally relevant recommendations. Participants stressed that the tool design should be simple, timesaving, and internet-independent. Electronic clinical decision support tools are capable of improving patient care and reduce antibiotic overuse, which may impact downstream antibacterial resistance. Future research should develop an eCDST for LRTI with local input and evaluate its impact on appropriate antibacterial use and patient outcomes.

## INTRODUCTION

Lower respiratory tract infections (LRTIs) are a top contributor to the global burden of disease, affecting millions of individuals annually and causing significant morbidity, mortality, and economic impact.^1^ In 2019, there were 488.9 million incident cases and 2.4 million deaths because of LRTIs globally.^1^^,^^2^ Lower respiratory tract infections were the leading cause of death among children under 5 years and the fifth cause of death among adults over 69 years.^2^ The impact of LRTIs is particularly pronounced in low- and middle-income countries (LMICs), which face challenges related to inadequate sanitation, malnutrition, air pollution from solid fuels, limited access to healthcare, and socioeconomic disparities.^1^^,^^3^

Lower respiratory tract infections have a wide variety of etiological agents, including bacteria, viruses, and fungi; however, a significant proportion of cases have unidentified etiological agents.^4^^,^^5^ Currently available diagnostic methods have limitations. Traditional microbiological, culture-based methods lack sensitivity, whereas newer, molecular-based tests only detect specific pathogens; therefore, even when diagnostic tests are used, a significant proportion of cases will still result in no etiological diagnosis.^6^ The advent of molecular diagnostics has shown that viruses are an important cause of LRTIs, and often more commonly identified than bacteria as the primary etiology in both children and adults.^4^^–^^7^ According to the United States EPIC (Etiology of Pneumonia in the Community) study, etiological agents were identified in 38% of the patients admitted with community-acquired pneumonia; viruses were detected in 30% of patients and bacteria in 14% of patients.^4^ However, etiology remained unidentified in over 50% of the cases despite important advances in the diagnostics in the last two decades.^4^^,^^5^^,^^8^

Antibacterial overuse in managing LRTIs is a global concern, contributing to the rise of antibacterial-resistant pathogens.^9^ In 2019, antibacterial-resistant infections were linked to almost 5 million deaths globally, with 1.27 million of these deaths directly attributed to drug resistance.^10^ If compared with all underlying contributors to mortality, antibacterial resistance would have ranked as the third leading cause of death in 2019.^10^ Antibacterial resistance affects nations of all regions and income levels, but poverty and inequality exacerbate the causes and consequences. Most antibacterial resistance-related deaths occur in LMICs because of a higher prevalence of infectious diseases, and limited access to diagnostic technology and newer-generation antibiotics.^10^^,^^11^ In Sri Lanka, overuse of antibacterials is a significant problem. In 2017, over half of all admitted hospital patients and almost all patients in intensive care wards were prescribed antimicrobials. About one-third of these antimicrobials were deemed potentially inappropriate.^12^ In the outpatient setting, more than 70% of patients presenting with acute respiratory tract infections receive antibacterial prescriptions, with many of these being for viral infections.^13^^,^^14^

In South Asia, inappropriate use of antibacterials is driven by factors across the social ecological framework.^15^ Therefore, multifaceted interventions to reduce overuse of antibacterials have been found to be effective and better than single initiatives in combating antimicrobial resistance.^9^ Some of the most effective measures include enforcing the policy preventing the over-the-counter sale of antibiotics, implementing antimicrobial stewardship programs, and using valid rapid point-of-care diagnostics.

Clinical decision support tools (CDSTs) may be another promising solution to address challenges in LRTI management and reduce inappropriate use of antibacterials, especially in low-resource settings.^16^^–^^18^ To support overburdened health professionals, CDSTs, which assist healthcare professionals in clinical decision-making, are becoming more common worldwide.^16^^,^^17^ Meta-analyses suggest that point-of-care tests, combined with an evidence-based diagnostic algorithm, can reduce inappropriate use of antibacterials in LMICs without compromising clinical outcomes.^19^ A Sri Lankan study of clinical algorithms and rapid influenza testing showed that clinical prediction tools and targeted diagnostics could potentially result in cost savings when considering the broader societal costs associated with antimicrobial resistance.^20^ Electronic CDSTs (eCDSTs), which are available on a website or mobile phone application, may be especially useful in low-resource clinical settings with high patient-to-physician ratios and limited diagnostic technology.^16^

The use of eCDSTs to support appropriate antibacterial prescribing for the management of LRTI remains unexplored in LMIC settings. Our qualitative study aimed to explore the potential of an eCDST to help healthcare providers in Sri Lanka effectively manage patients with LRTIs.

## MATERIALS AND METHODS

This was a qualitative study to explore the potential of eCDSTs to manage adult patients with LRTIs in Sri Lanka. Individual semi-structured interviews were conducted with 15 physicians from six hospitals between March and August 2023. Ethical clearance for this study was obtained from Ethics Review Committee, Faculty of Medicine, University of Ruhuna, Sri Lanka (Reference No. 15.02.2018.3.13), obtained on February 16, 2023.

### Setting and participants.

We used convenience sampling to identify six public hospitals in the Western and Southern regions of Sri Lanka to participate in the study. Study sites included three tertiary care hospitals (approximately 1,000–3,000 beds), one district general hospital (1,000 beds), and two base hospitals (360–400 beds). The participating facilities were all public hospitals with microbiological laboratories and radiology facilities, and provide all medications, diagnostics, and care to patients free of charge. Tertiary care hospitals in Sri Lanka have higher bed capacity, radiological and laboratory capabilities, and physicians than district general and base hospitals.

To ensure broad representation of provider characteristics, we selected four consultant physicians, five post-Doctor of Medicine (MD) senior registrars, and four trainee medical registrars from the six hospitals.

### Procedures.

Participants provided written informed consent to participate in the study, including permission for audio recording. In-depth interviews were conducted by three investigators (W.D.Z., L.G.T., D.P.), all of whom had MD training and clinical experience in Sri Lanka. The interviewers were trained in qualitative methods and were supervised by a qualitative methods expert (M.H.W.). The interviewers used a semi-structured interview guide, which was developed by the multinational study team. The guide included open-ended questions and follow-up probes across three domains: 1) experience treating LRTI in the inpatient setting in Sri Lanka, 2) experience and opinions on eCDSTs, and 3) opinions and feedback on an eCDST to manage pneumonia and other LRTIs in adults.

Interviews were conducted in English and lasted approximately 45 minutes. The interviews were conducted in-person (*n* = 7) or via video-conferencing (*n* = 8), depending on the participant’s preference. All interviews were audio-recorded and transcribed verbatim.

## STATISTICAL ANALYSES

Data analysis was conducted iteratively using rapid turnaround qualitative analysis, an efficient and rigorous qualitative approach that was developed for implementation of science research.^21^ The analysis process involved summary and familiarization with individual interviews, identification of emerging themes across interviews, coding to group data, and synthesis and interpretation of the dataset. As data were being collected, we wrote “episode profiles” to summarize each interview. The episode profiles were three to four pages each and followed the template of the interview guide. These summaries were produced by one study team member (J.G.), and both the transcripts and summaries were reviewed by another team member with qualitative expertise (M.H.W.). Any discrepancies were resolved through discussion. The episode profiles allowed us to recognize and record emerging themes in each individual piece of data, and to re-strategize our data collection process as needed to meet our stated objective.^22^ The episode summaries were then uploaded into NVIVO (Version10.2, QSR International, Burlington, MA) and coded to group-emerging content in each domain.^23^^,^^24^ We generated code reports for four domains (interest in an eCDST, tool capabilities, features to consider in the development of the tool, and considerations for tool design) and discussed the coded content among the group to identify emerging themes. The themes were developed through consensus to ensure that each theme accurately represented the coded extracts and that themes were distinct from one another. The data were then synthesized into an analytic memo that incorporated examples and quotes to illustrate each theme.

## RESULTS

We enrolled 15 physicians, including 10 males and five females. The average age of participants was 38 years, with an average of 11.6 years of clinical experience (range 4–25 years). Among the recruited physicians, 11 were from tertiary care hospitals, two were from district general hospitals, and two were from base hospitals. Notably, six out of 15 participants were consultant physicians with 14–25 years of clinical experience, and five were post-MD senior registrars with 5–12 years of clinical experience. Four participants were medical registrars (pre-MD) with clinical experience ranging from 4 to 5.5 years. Designation, gender, hospital type, and the years of clinical experience of the participants are illustrated in Table 1.

**Table 1:** Participant designation, gender, hospital type, and years of clinical experience (*n* = 15)

Designation	Sex	Hospital Type	Years of Clinical Experience
Consultant	Male	Base hospital	24
Consultant	Male	Base hospital	20
Consultant	Male	District general hospital	22
Consultant	Male	District general hospital	25
Consultant	Male	Teaching hospital	15
Consultant	Female	Teaching hospital	14
Senior registrar	Male	Teaching hospital	12
Senior registrar	Female	Teaching hospital	5
Senior registrar	Male	Teaching hospital	6
Senior registrar	Male	Teaching hospital	7
Senior registrar	Female	Teaching hospital	5
Registrar	Female	Teaching hospital	5.5
Registrar	Male	Teaching hospital	5
Registrar	Female	Teaching hospital	4
Registrar	Male	Teaching hospital	4

The interviews with physicians revealed broad interest in an eCDST to manage pneumonia and other LRTIs in adult patients, and identified themes related to tool capabilities, features to consider in the development of the tool, and considerations for tool design (Table 2). We did not observe any differences in the themes that emerged by participants’ years or experience or other demographic variables.

**Table 2:** Themes related to tool capabilities, features to consider in the development of the tool, and considerations for tool design

Domains	Themes
Desires for tool capabilities	Information about the pathogen Treatment recommendations Severity of LRTI Monitoring of patient progress
Features important to consider in the development of the eCDST	Validated algorithms Regional specificity Seasonality Inclusion of patient’s risk factors Scalability Importance of updated and locally relevant recommendations
Design characteristics of the tool	Easy to use Responsive to local resources

ECDST = electronic clinical decision support tool; LRTI = lower respiratory tract infection.

### Interest in an eCDST for managing LRTIs.

All providers expressed enthusiasm for incorporating eCDSTs for managing LRTIs into their practice, as they felt eCDSTs would provide objective, evidence-based guidance for clinical management of patients. One physician stated:

*“Because it incorporates the probability, probable organisms, and that can be useful in our clinical decision-making” (male clinician, 15 years of experience).*

Three participants emphasized that an eCDST could be useful, but should not replace clinical judgement, and that the utility of the tool depended on the accuracy of the algorithm behind the tool. Another participant stated that he would not use this tool with all patients, but rather only with patients where he felt clinical uncertainty.

*“If I decide to use a tool for a patient, it means that there is something that I’m not confident to make the diagnosis. If I am going to use the tool, I agree to feed the information that the tool needs. Otherwise, I do not use the tool. But for honest purposes, considering the time consumption and the cost, I won’t apply the tool for all the patients” (male clinician, 6 years of experience).*

When evaluating the significance of eCDST in clinical practice, the percentage of providers who were entirely favorable toward it and the proportion expressing some reservations were nearly identical in quantity and years of experience.

### Desires for tool capabilities.

When providers were asked about their expectations for the eCDST, four themes emerged regarding desired capabilities.

#### Information about the pathogen.

Participants wanted the eCDST to provide information to support whether the pathogen was bacterial or viral. One participant noted that they would like to know the probability that a certain pathogen was causing the infection. Two participants noted that they would like to know whether the pathogen was community- or hospital-acquired.

#### Treatment recommendations.

Participants wanted the eCDST to provide recommendations for treatment and management, such as whether the patient should be admitted, whether antibiotics or antivirals should be prescribed, and if so, the specific medication and route of administration. Participants emphasized that the tool should provide detailed insights into whether the recommended medication might interact adversely with the patient’s existing treatments. One participant was an exception to this, stating that he did not wish to receive information about treatment recommendations because he didn’t “want to depend on an electronic device.”

Clinicians demonstrated a similar willingness to comply with the tool’s guidance on treatment recommendations, irrespective of their experience, voicing their concerns about the need to factor in the patient’s entire clinical profile when making these decisions, rather than depending exclusively on the tool.

#### Severity of LRTI.

Participants wanted the eCDST to provide a score on severity of LRTI that could be linked with treatment recommendations. Being able to quantify severity of illness would support clinical decisions related to monitoring escalation of therapy.

#### Monitoring of patient progress.

Several physicians felt that the eCDST should be able to be used longitudinally to monitor patient progress and inform clinical decision-making to respond to clinical changes.

“If we apply in day two, based on the day one value and when I apply the second time, day two of the app is telling me patient is clinically improving, that would be relying factor for me. That would be helpful” (male clinician, 6 years of experience).

### Features important to consider in the development of the eCDST.

When providers were asked about features that would be important to consider during eCDST development, six themes emerged.

#### Validated algorithms.

Participants indicated that the tool should be scientifically grounded and validated so that they have confidence in the output.

“Need to kind of retest it and in several different centers around the country with varied patterns of respiratory infections to make sure it works everywhere the same way” (female clinician, 5 years of experience).

Additionally, participants emphasized the importance of the availability of supporting evidence such as user-review or institutional agreement on following the tool’s recommendations.

#### Regional specificity.

Participants indicated that the tool should consider the prevalence of pathogens in the local geographic area and identify the most common pathogens, as the prevalence could fluctuate across different regions.

#### Seasonality.

Participants indicated that the tool should be responsive to seasonal variations and outbreaks, and should incorporate that data into the algorithm.

#### Inclusion of patient’s risk factors.

Physicians emphasized the critical importance of having a tool that considers the patient’s risk factors such as age and comorbidities.

“It should be based on the patient’s clinical symptoms and complications and their comorbidities and any other risk factors to develop those respiratory tract infections. And any underlying chronic lung diseases, it should include those factors as well,” (female clinician, 5 years of experience).

#### Scalability.

Participants indicated that the tool should be usable across all government hospitals in the country. Ensuring widespread access and usage of the tool would facilitate its integration into clinical practice. They recommended the development of a common tool that could be easily adopted and used across all hospitals, facilitating streamlined management of respiratory infections.

*“So, my advice is having a tool, a common tool would be better so that it will be used all over most of the government hospitals. So the management would be much easier, or it could much more augmented with the tool” (male clinician, 25 years of experience).*

#### Importance of updated and locally relevant recommendations.

Participants indicated that the tool should be grounded in treatment options that are available locally.

*“What antibiotic is there or what investigation is available today will not be available tomorrow. So that information has to be fitted at some sort of a kind of a database we can correlate with the laboratories and pharmacies, and ask them what are the available drugs, and it should be readily available in our sector. It should be fit into this app what are available drugs and investigations” (male clinician, 12 years of experience).*

### Design characteristics of the tool.

When asked for anticipated characteristics of the tool’s design, two themes emerged.

#### Easy to use.

Participants emphasized that the tool should be simple, without excessive inputs, and be easy to use.

*“It should be very simple. At the same time, there should be enough space to give enough information. We should be able to feed all the enough information to get a proper decision. But it should be simple. If it is complicated, we won’t use it. Just yes/no questions would be better” (male clinician, 4 years of experience).*

#### Responsive to local resources.

Physicians felt that the tool should only require inputs (e.g., laboratory results) that are available in all healthcare facilities. Also, it should be able to work in an offline mode, since internet is not consistently available in all facilities.

## DISCUSSION

The findings from this qualitative study underscore the potential of eCDSTs to improve the management of LRTIs in low-resource settings such as Sri Lanka. Participants expressed a clear interest in integrating eCDSTs into their practice, recognizing the benefits such tools could offer in promoting evidence-based decision-making, but at the same time, they stressed the importance of prioritizing clinical judgment over algorithmic recommendations. The interviews revealed insights into the necessary attributes and design considerations for these tools to be effective and acceptable to practitioners. Participants suggested that the eCDST should include information on the causative pathogen, suggestions for treatment, and system for grading the severity of LRTIs. They suggested that the tool’s design should incorporate regional specificity, seasonality, and validated algorithms. Additionally, the feedback emphasized the need for the eCDST to be simple, time-efficient, and capable of functioning without internet dependency. These considerations are crucial in developing a tool that is both practical and impactful in enhancing patient care and addressing the challenge of antibiotic resistance.

Efficient management of LRTIs relies on the differentiation between infectious and noninfectious causes, as well as the identification of the microbial agent, if applicable. Lack of adequate diagnostics is a major constraint to the optimum management of LRTIs in low-resource settings.^25^^–^^27^ In high-resource countries, tests for specific pathogens and biomarkers help clinicians decide if antibacterials are clinically warranted; however, the consistent accessibility of such tests remains scarce in regions with limited resources.^28^ The coronavirus disease 2019 (COVID-19) pandemic has underscored the importance of strong diagnostic capacity, highlighting the importance of rapid point-of-care diagnostic tests that are typically unavailable in LMICs.^29^ In resource-poor settings, the lack of access to accurate and timely diagnostic testing for patients with LRTIs often forces healthcare providers to rely solely on clinical assessments for treatment decisions, resulting in the inappropriate use of antibacterials and poor patient outcome.^26^ A study done in Southwestern Uganda showed that using simple, rapid tests for specific pathogens and biomarkers was feasible in resource-limited settings and could help improve the management of respiratory illnesses and antibiotic stewardship.^28^ Successful case studies of timely integration of molecular diagnostics into clinical practice have led to expedited diagnosis, enhanced disease management, and improved control of infections, such as COVID-19 and Ebola in LMICs.^30^^–^^32^

Electronic clinical decision support tools are emerging as a solution to improve the management of LRTIs and other infections in low-resource settings by integrating patient health information and point-of-care diagnostics with evidence-based clinical protocols.^17^^,^^18^ Systematic reviews have shown that eCDSTs positively impact antibacterial use in LRTIs, with 15 out of 22 studies reporting statistically significant improvements in prescription practices and adherence to guidelines, thereby promoting more judicious use of antibacterials.^33^ In the United States, a procalcitonin-based decision algorithm to guide antibacterial prescription for hospitalized sepsis and LRTI patients resulted in shorter length of stay, reduced antibacterial use, fewer mechanical ventilation days, and fewer patients with *Clostridium difficile* and antibacterial-resistant infections compared with standard care. Furthermore, procalcitonin-guided healthcare resulted in cost savings of $25,611 (49% reduction from standard care) for sepsis and $3,630 (23% reduction) for LRTI, on average per patient.^34^ The use of an evidence-based eCDST can also enhance LRTI management by facilitating the timely switch from parenteral to oral antibiotics based on clinical and biochemical indicators, leading to improved patient outcomes and cost savings.^35^^,^^36^ Overall, eCDSTs provide a framework for managing LRTIs by combining diagnostic precision, comprehensive treatment plans, and effective medication management, thereby enhancing the overall quality of care, reducing antibacterial overuse, and improving patient satisfaction. The unanimous interest among Sri Lankan clinicians in combining an eCDST with clinical judgment to manage LRTIs provides a strong foundation for implementing this approach into clinical practice in Sri Lanka. Clinicians can use eCDSTs to access additional information and evidence-based recommendations, aiding in the management of LRTIs while still relying on their clinical judgment for final decisions. ECDSTs hold significant promise for improving healthcare outcomes in Sri Lanka by addressing critical challenges such as the scarcity of clinicians, limited resources, and high disease burden. In African countries, where healthcare systems often struggle with these issues, the implementation of CDSTs has shown to be effective, efficient, and reliable in diagnosing and managing diseases, particularly pediatric and maternal conditions.^37^^,^^38^

Developing an eCDST for managing LRTIs requires consideration of several critical factors. The interviewed clinicians identified the main desired tool capabilities as guidance on etiology, suggestions for treatment, and a system to grade the severity of LRTIs. ECDSTs can significantly enhance the differentiation between viral and bacterial etiologies in LRTIs for appropriate antibacterial use; furthermore, they can provide detailed information about the causative pathogen guiding the treatment.^39^^,^^40^ According to a randomized controlled trial across six LMICs, a package of point-of-care tests and a clinical algorithm for outpatient acute fever case management reduced by 30% unnecessary antibacterial prescribing without negatively affecting clinical outcomes by differentiating viral from bacterial etiology.^41^ The primary factors highlighted by the providers in the development of the eCDST included considering regional specificity, seasonality, and using validated algorithms to ensure the effectiveness and dependability of the tool. Regional specificity is crucial as it allows the eCDST to account for geographical variations in disease prevalence, pathogen distribution, and patient demographics, which can significantly impact treatment outcomes. Seasonality is another critical factor, as seasonal changes can influence the prevalence of specific pathogens that cause LRTIs. Incorporating seasonal data into an eCDST can help predict outbreaks and adjust treatment protocols accordingly. Validated algorithms are essential for the reliability of eCDSTs. By integrating these elements into the development of eCDSTs for LRTIs, healthcare providers can achieve more accurate diagnoses, personalized treatments, and ultimately, better patient outcomes.^33^ Sri Lanka faces significant challenges because of high patient volumes and limited internet connectivity in the hospital setting, making user-friendly and internet-independent tools crucial.

There has been little published research to date on the effectiveness of eCDSTs in clinical practice, particularly in low-resource settings with promising results.^42^^,^^43^ There is limited evidence on using CDSTs in Sri Lanka, and though providers see the usefulness of CDSTs in clinical practice, they emphasize the importance of scientific validity before incorporating them into their practice.^20^ The reluctance of providers to make decisions based on the tool’s recommendations underscores the need for a comprehensive evaluation of its effectiveness and impact at a larger scale in low-resource settings.

### Limitations.

This study has several limitations that must be acknowledged. Firstly, the sample size was relatively small, which may limit the generalizability of the findings and may have prevented us from seeing any differences in themes by participant demographic characteristics. Furthermore, selecting physicians from hospitals with greater resources may not fully represent the national clinical setting. Moreover, discussion of the eCDST was largely theoretical. The real potential and practical utility of the eCDST can be best assessed with a working prototype in a clinical setting. Future research should address these limitations by including larger, more diverse samples and by evaluating the eCDST with a functional prototype in real clinical environments.

## CONCLUSION

Electronic clinical decision support tools are innovative tools that have the potential to improve judicious diagnosis and treatment of LRTIs in Sri Lanka, reducing inappropriate antibacterial use and improving patient outcomes. For maximum effectiveness, these tools should be developed with substantial local input to ensure they are relevant and user-friendly. Research is required to assess the impact of eCDSTs on antibacterial use and patient outcomes, ensuring ongoing improvement and optimal integration into the healthcare system.
